# PolyDeep Advance 1: Clinical Validation of a Computer-Aided Detection System for Colorectal Polyp Detection with a Second Observer Design

**DOI:** 10.3390/diagnostics15040458

**Published:** 2025-02-13

**Authors:** Pedro Davila-Piñón, Teresa Pedrido, Astrid Irene Díez-Martín, Jesús Herrero, Manuel Puga, Laura Rivas, Eloy Sánchez, Sara Zarraquiños, Noel Pin, Pablo Vega, Santiago Soto, David Remedios, Rubén Domínguez-Carbajales, Florentino Fdez-Riverola, Alba Nogueira-Rodríguez, Daniel Glez-Peña, Miguel Reboiro-Jato, Hugo López-Fernández, Joaquín Cubiella

**Affiliations:** 1 Research Group in Gastrointestinal Oncology Ourense (REGGIOu), Hospital Universitario de Ourense, 32005 Ourense, Spain; pedro.davila@iisgaliciasur.es (P.D.-P.); teresapedridofdez@gmail.com (T.P.); astrid.diez@iisgaliciasur.es (A.I.D.-M.); 2 Fundación Pública Galega de Investigación Biomédica Galicia Sur, Hospital Universitario de Ourense, SERGAS, 32005 Ourense, Spain; 3 Department of Gastroenterology, Hospital Universitario de Ourense, Centro de Investigación Biomédica en Red de Enfermedades Hepáticas y Digestivas (CIBEREHD), 32005 Ourense, Spain; jesus.miguel.herrero.rivas@sergas.es (J.H.); manuel.puga.gimenez.de.azcarate@sergas.es (M.P.); laura.rivas.moral@sergas.es (L.R.); eloy.sanchez.hernandez@sergas.es (E.S.); sara.zarraquinos.martinez@sergas.es (S.Z.); noel.pin.vieito@sergas.es (N.P.); pablo.vega.villaamil@sergas.es (P.V.); santiago.soto.iglesias@sergas.es (S.S.); david.rafael.remedios.espino@sergas.es (D.R.); 4 Department of IT, Hospital Universitario de Ourense, 32005 Ourense, Spain; ruben.dominguez.carbajales@sergas.es; 5 Department of Computer Science, Escuela Superior de Ingenieria Informática (ESEI), CINBIO, University of Vigo, 32004 Ourense, Spain; riverola@uvigo.gal (F.F.-R.); alnogueira@uvigo.gal (A.N.-R.); dgpena@uvigo.gal (D.G.-P.); mrjato@uvigo.gal (M.R.-J.); 6 Next Generation Computer Systems Group (SING) Research Group, Galicia Sur Health Research Institute (IIS Galicia Sur), 32004 Ourense, Spain; 7 Instituto de Investigação e Inovação em Saúde (i3S), Universidade do Porto, Rua Alfredo Allen 208, 4200-135 Porto, Portugal

**Keywords:** colonoscopy, computer-aided detection, detection, polyps, performance, screening colonoscopy, FIT, surveillance

## Abstract

**Background:** PolyDeep is a computer-aided detection and characterization system that has demonstrated a high diagnostic yield for in vitro detection of colorectal polyps. Our objective is to compare the diagnostic performance of expert endoscopists and PolyDeep for colorectal polyp detection. **Methods:** PolyDeep Advance 1 (NCT05514301) is an unicentric diagnostic test study with a second observer design. Endoscopists performed colonoscopy blinded to PolyDeep’s detection results. The main endpoint was the sensitivity for colorectal polyp (adenoma, serrated or hyperplastic lesion) detection. The secondary endpoints were the diagnostic performance for diminutive lesions (≤5 mm), neoplasia (adenoma, serrated lesion) and adenoma detection. **Results:** We included 205 patients (55.1% male, 63.0 ± 6.2 years of age) referred to colonoscopy (positive faecal immunochemical occult blood test = 60.5%, surveillance colonoscopy = 39.5%). We excluded eight patients due to incomplete colonoscopy. Endoscopists detected 384 lesions, of which 39 were not detected by PolyDeep. In contrast, PolyDeep predicted 410 possible additional lesions, 26 of these predictions confirmed by endoscopists as lesions, resulting in a potential 6.8% detection increase with respect to the 384 lesions detected by the endoscopists. In total, 410 lesions were detected, 20 were not retrieved, five were colorectal adenocarcinoma, 343 were colorectal polyps (231 adenomas, 39 serrated and 73 hyperplastic polyps), 42 were normal mucosa and 289 were ≤5 mm. We did not find statistically significant differences between endoscopists and PolyDeep for colorectal polyp detection (Sensitivity = 94.2%, 91.5%, *p* = 0.2; Specificity = 9.5%, 14.3%, *p* = 0.7), diminutive lesions (Sensitivity = 92.3%, 89.5%, *p* = 0.4; Specificity = 9.8%, 14.6%, *p* = 0.7), neoplasia (Sensitivity = 95.2%, 92.9%, *p* = 0.3; Specificity = 9.6%, 13.9%, *p* = 0.4) and adenoma detection (Sensitivity = 94.4%, 92.6%, *p* = 0.5; Specificity = 7.2%, 11.8%, *p* = 0.2). **Conclusions:** Expert endoscopists and PolyDeep have similar diagnostic performance for colorectal polyp detection.

## 1. Introduction

Colorectal cancer (CRC) is the third most frequent cancer and the second leading cause of cancer-related mortality worldwide [1,2]. The development of precursor lesions, including adenomas and serrated lesions, plays a critical role in its progression [3]. In recent years, public health systems have implemented CRC screening programmes for average-risk individuals aged 50–75 years to reduce both its incidence and mortality [1,2,3,4]. However, poor quality of colonoscopy—induced by inadequate bowel preparation, insufficient mucosal exposure or endoscopist fatigue—can lead to missed precursor lesions and the subsequent development of post-colonoscopy CRC [5].

Artificial intelligence (AI) is a research field focused on developing machines capable of performing tasks that typically require human intelligence. One of its most significant subfields is Machine Learning (ML), where AI models learn to execute tasks for which they were not explicitly programmed [2]. Within ML, Deep Learning (DL) represents a specialised subset of ML techniques and relies on large artificial neural networks, usually with a high number of layers, enabling the automatic learning of complex features [6]. The success of DL techniques in recent years, surpassing the performance of classical ML methods, has triggered a renewed surge of interest in AI. In the medical field, this is reflected in the growing number of AI-based medical devices authorised by the FDA [7]. Notably, more than 770 devices have been approved in the past decade, a stark increase compared to the 27 approvals in previous years [7]. Among the most impactful AI-based medical devices, particularly in colonoscopy, are Computer-Aided Diagnosis (CAD) systems. These systems support medical professionals during procedures to enhance decision-making by extracting relevant features from colorectal lesion images, enabling both detection (CADe) and characterisation (CADx) [2,4,8,9]. The worldwide integration of CAD systems into medical image analysis represents a revolution in routine clinical practice. In colonoscopy, CADe has been shown to improve procedure quality by enhancing key performance indicators such as the adenoma detection rate (ADR), polyp detection rate (PDR) and adenoma miss rate (AMR) [10]. A common study design to evaluate the impact of CADe on polyp detection is based on a second observer, with the endoscopist blinded to CADe outputs [11,12,13].

PolyDeep is a DL-based CADe/x system designed to detect and characterise colorectal lesions [14,15,16,17,18]. The system integrates a YOLOv3 detection network, coupled with an object tracking algorithm and a ResNet50 classification network [14,17,18]. PolyDeep was developed using transfer learning techniques, fine-tuning models (ResNet50 based on ImageNet and YOLOv3 on PASCAL VOC) with still images of colorectal lesions extracted from colonoscopy videos [14,17,18]. As part of the development of PolyDeep, we created PIBAdb (Polyp Image BAnk database), a comprehensive database of images and videos of colorectal lesions manually annotated by expert endoscopists, which is currently available in the biobank of the Instituto de Investigación Sanitaria Galicia-Sur [19]. Prior to clinical validation, we enhanced the detection and characterization models by incorporating new datasets from the project and adjusting several configuration parameters to optimise the CADe/x system for a real colonoscopy procedure [16]. Specifically, the detection model was improved through fine-tuning with non-polyp images to reduce false positives (included in PIBAdb), while the characterisation model was retrained with new polyp images obtained during the clinical validation phase (although these new images are not already included in PIBAdb). The aim of this study is to evaluate and compare whether the diagnostic performance of PolyDeep is better than that of the expert endoscopists in detecting colorectal lesions in a population-based colorectal cancer screening programme.

## 2. Materials and Methods

### 2.1. PolyDeep Advance 1: Study Design

PolyDeep Advance 1 (NCT05514301) is a single-centre, prospective diagnostic test study with a second observer design. The research protocol was approved by the local institutional review board (2022/067).

### 2.2. Inclusion and Exclusion Criteria

We enrolled patients referred to colonoscopy within the Galician CRC screening programme in Ourense, Spain; either after a positive faecal immunochemical test (FIT) or as a surveillance colonoscopy after the resection of advanced colorectal lesions. All study participants provided written informed consent. We excluded individuals with a personal history of CRC, colonic resection or any syndrome predisposed to CRC, such as hereditary syndromes or serrated polyposis syndrome. For the analysis, we excluded participants when caecal intubation was not achieved or with insufficient bowel cleansing (defined as Boston Bowel Preparation Scale < 2 in any segment or <6 overall).

### 2.3. Clinical Setting

The endoscopists performed the colonoscopy in the conventional clinical setting, blinded to the CADe/x system. A second observer attended the colonoscopy viewing the PolyDeep images on a separate screen. When PolyDeep identified a potential lesion, it triggered a visual alert in the form of a red border around the screen, which was activated when the CADe consistently tracked one or more potential polyps (also indicated by bounding boxes) across several video frames. If PolyDeep identified a possible lesion that was not detected by the endoscopists, the second observer alerted the endoscopists for confirmation. All detections rejected by the endoscopists (i.e., bubbles, stool, normal mucosa and undefined artefact) were classified as false positives. Thus, the endoscopist was responsible for confirming the CADe detections. The second observer recorded the detections and characterisation made by both the endoscopists and PolyDeep in a log sheet form.

Additionally, the endoscopists classified lesions according to the Narrow Band Imaging International Colorectal Endoscopic (NICE) classification [20], categorising them as adenomas (NICE II) or non-adenomas (NICE I). PolyDeep is trained to detect and characterise colorectal lesions as neoplasms, including adenomas and serrated lesions, or non-neoplasms, such as hyperplastic lesions. During colonoscopy, the CADe/x system identified lesions by bounding boxes around them and characterised them as neoplasia if the probability assigned to the bounding box was greater than 50% [14,17,18].

### 2.4. Study Endpoints

The gold standard for this study was histology. Lesions without histology or cases of CRC were excluded from the analysis. The primary endpoint of the trial was the detection of colorectal polyps, including adenomas, serrated lesions and hyperplastic lesions. The secondary endpoints included the detection of adenomas, serrated lesions (i.e., sessile serrated lesions or traditional serrated adenomas), neoplasia (i.e., adenomas or serrated lesions), diminutive (≤5 mm) polyps and advanced colorectal polyps (i.e., adenomas or serrated lesions ≥ 10 mm, adenomas with villous histology or high-grade dysplasia, or serrated lesions with dysplasia).

### 2.5. Data Collection and Study Population

We designed the electronic case report form (eCRF) using the RedCap platform at the Galicia-Sur Health Research Institute (IIS Galicia-Sur) (https://redcap.tic1-iisgaliciasur.es/ accessed on 26 April 2024). The eCRF captured data on demographics, colonoscopy and lesion characteristics (including location, size and histology). Additionally, we included information on the optical diagnosis and the histology predicted by the endoscopists and the CADx.

### 2.6. Sample Size Calculation

We calculated the sample size based on the assumption of 90% sensitivity for the endoscopists and 95% sensitivity for PolyDeep, with a beta error of 20%, an alpha error of 5% and an estimated 5% rate of incomplete colonoscopies [21]. Based on these assumptions, we determined a sample size of 205 patients, with a mean of 2.5 lesions detected per procedure, to evaluate a total of 487 lesions in the study.

### 2.7. Statistical Analysis

We performed a descriptive analysis of the study population. Qualitative variables are presented as absolute frequencies and percentages, while quantitative variables are presented as means and standard deviations. For the primary and secondary endpoints, we calculated the diagnostic performance metrics (i.e., sensitivity, specificity, positive and negative predictive values, odds ratio and Area Under the Curve-AUC) for endoscopists and PolyDeep. The comparison between endoscopists and PolyDeep was performed using the McNemar test. In all categories, we conducted sub analyses by size (≤5 mm or >5 mm), morphology (protruded or non-protruded) and location (right colon or left colon). Additionally, the precision of endoscopists and PolyDeep in the characterisation of adenomatous and neoplastic lesions, respectively, was determined. We used the statistical software R version 4.3.0 (The R Foundation for Statistical Computing, Institute for Statistics and Mathematics, Vienna, Austria) for statistical analysis.

## 3. Results

### 3.1. Population Description and Main Endpoints Results

Between February 2023 and April 2023, we recruited 205 patients (55.1% male, 63 ± 6.2 years of age) who participated in the Galician population-based CRC screening programme. We included patients who underwent colonoscopy after a positive FIT (60.5%) or as endoscopic surveillance (39.5%). We excluded eight patients from the study: seven due to insufficient bowel cleansing or no caecal intubation and one due to a personal history of CRC and previous colonic resection.

**Figure 1:**
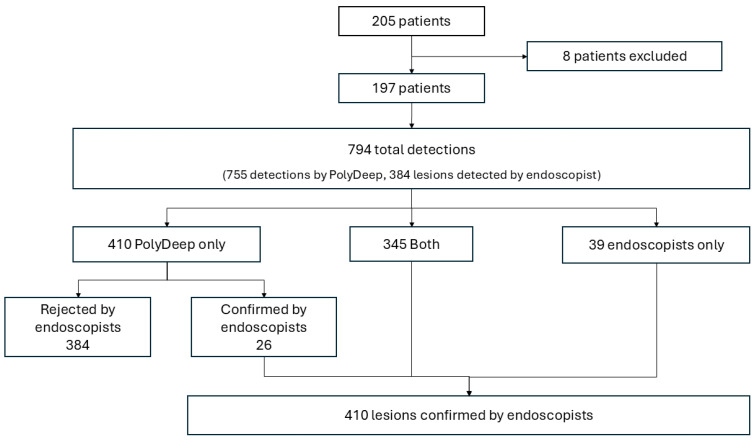
Flowchart of the study.

Overall, the CADe system and endoscopists performed 794 detections, including 410 lesions and 384 false positives identified by PolyDeep. The endoscopists detected 384 lesions, of which PolyDeep missed 9.51%. PolyDeep detected 371 lesions, of which the endoscopists initially missed 6.34% (Figure 1). Using the endoscopists’ detections as the reference for lesion identification (without considering histological analysis), the sensitivity for endoscopists was 93.7% ((384/410) × 100), while the sensitivity for PolyDeep was 90.5% ((371/410) × 100).

Table 1 presents the characteristics of the lesions according to the observer (both, endoscopists or PolyDeep). Overall, the histology of the identified polyps was adenomas (56.34%), serrated lesions (9.51%), hyperplastic lesions (17.80%), normal mucosa (10.24%), non-retrieved polyps (4.88%) and CRC (1.22%). Most of the lesions were small and located in the right colon and exhibited a protruded morphology. Polyps detected during the study had a mean size of 5.1 ± 4.9 mm.

### 3.2. Diagnostic Performance for Polyp Detection

We excluded 25 lesions from the diagnostic performance analysis: five cases of CRCs and 20 lesions without histology. Table 2 shows the diagnostic performance of the endoscopists and PolyDeep for polyp detection. No statistically significant differences were found in sensitivity (94.2%, 91.5%, *p* = 0.2), specificity (9.5%, 14.3%, *p* = 0.7) or AUC (0.518 vs. 0.529, *p* = 0.8) between endoscopists and PolyDeep. The 91.5% sensitivity achieved by the CADe system in the clinical evaluation surpasses the 89.91% (87.20–91.94%) sensitivity obtained by the initial detection model in a video-based evaluation [17].

In the sub-analysis by size, we found no statistically significant differences between the endoscopists and PolyDeep. Regarding lesion morphology, although diagnostic performance did not differ in protruded lesions, endoscopists demonstrated higher sensitivity than PolyDeep in the detection of non-protruded lesions (94.5% vs. 82.4%, *p* < 0.05), with no differences in specificity (11.8% vs. 17.6%, *p* = 1.0).

In the secondary analysis, we compared the diagnostic performance of endoscopists and PolyDeep according to the lesion histology. As shown in Table 3, no differences in sensitivity, specificity or AUC (i.e., neoplasia: 0.523 vs. 0.534, *p* = 0.7) were found in any of the categories evaluated. Data on the diagnostic odds ratio, as well as the positive and negative predictive values, are also presented in Table 3.

### 3.3. Diagnostic Performance for Polyp Characterisation

We calculated the diagnostic performance of PolyDeep and endoscopists for the characterisation of neoplastic/non-neoplastic and adenomatous/non-adenomatous lesions when histology was available (*n* = 385). The endoscopists provided an optical diagnosis for all 385 lesions (100%), classifying 149 as NICE I (38.2%), 236 as NICE II (60.5%) and five as NICE III (1.3%). In comparison, PolyDeep achieved an optical diagnosis for 329 lesions (85.5%), classifying 259 (78.7%) as neoplastic (with a probability > 50%) and 70 (21.3%) as non-neoplastic (with a probability ≤ 50%).

For adenoma histology prediction (NICE II), the sensitivity, specificity and AUC of endoscopists were 76.6% (95% CI 70.5–81.8%), 61.7% (95% CI 53.5–69.3%) and 0.693 (95% CI 0.645–0.740), respectively. In contrast, for neoplastic histology prediction, PolyDeep exhibited a sensitivity of 82.6% (95% CI 77.1–87.1%), a specificity of 32.2% (95% CI 22.8–43.2%) and an AUC of 0.671 (95% CI 0.607–0.734)

## 4. Discussion and Conclusions

PolyDeep is a CADe/x system that has demonstrated its ability to detect and characterise polyps in an in vitro study [16]. In this prospective diagnostic test study, PolyDeep increases the number of lesions detected during colonoscopy. When compared PolyDeep to blinded expert endoscopists, with histology as the gold standard, we found no significant differences in the detection of polyps, adenomas, neoplasia or advanced colorectal lesions. These findings suggest the potential of PolyDeep as a clinical support tool for colonoscopy procedures.

For the clinical validation of PolyDeep, we adopted a widely validated study design [11,12,13,22,23], which enabled us to evaluate the impact on endoscopic diagnostic performance. Our results are comparable to other studies that used a second observer design [12,13,23]. The use of CADe systems in colonoscopy improves quality indicators (such as ADR, PDR or AMR) by increasing lesion detection. In our study, the simultaneous detection of lesions by endoscopists and PolyDeep was lower than in a pilot study, where endoscopists and the CAD-ARTIPOD detected more lesions (84.1% vs. 94.7%) [13]. However, PolyDeep increased the percentage of lesions detected by 6.8%, which is higher than other studies with similar designs, which reported a 4% increase [12]. This increase in polyp detection can directly improve the quality of colonoscopy. Thus, using second observers (e.g., trainees or experienced nurses) or CADe systems (acting as standardised second observers) could potentially lead to improvements in ADR and PDR [11,22,24,25]. Although some meta-analyses highlight no statistically significant differences between the use of CADe systems and second observers in terms of ADR or adenomas per colonoscopy (APC) [22], evaluating the effect on quality indicators was beyond the scope of our study. Two ongoing multicentre randomised controlled trials (PolyDeep Advance 2, NCT05512793, and PolyDeep Advance 3, NCT05513261) aim to evaluate AMR and ADR.

Our study was conducted within the context of CRC screening colonoscopies. This setting may limit some of our findings, as screening endoscopists are highly experienced in detecting small lesions [26]. Despite the similarities with other studies, the sensitivity of endoscopists and PolyDeep in our study (94.2% and 91.5%) was lower than that observed in a pilot study, where expert endoscopists and the CAD-ARTIPOD system achieved sensitivities of 98.2% and 96.5%, respectively [13]. The CAD-ARTIPOD study, a multicentric trial using the same CADe system as the pilot study, reported a sensitivity of 94.6% for endoscopists and 96.1% for CAD-ARTIPOD using the endoscopists identification as reference standard and not considering the histological analysis [12,13,23]. In our study, the sensitivity of endoscopists (93.7%) and PolyDeep (90.5%) were comparable to those observed in the CAD-ARTIPOD study not considering histological analysis [12,13,23]. When we considered histology as the gold standard, the diagnostic performance (sensitivity) of endoscopists and CAD-ARTIPOD (94.9% vs. 96.0%) was superior to that of endoscopists and PolyDeep (94.2% vs. 91.5%) [12,23]. We evaluated the diagnostic performance of endoscopists and PolyDeep separately; however, in real clinical practice, both work in tandem (i.e., endoscopists assisted by CADe/x systems). Several studies compared the diagnostic performance of conventional colonoscopy to CADe-assisted colonoscopy [27,28], highlighting the importance of optimising collaboration between endoscopists and AI systems to achieve maximum diagnostic performance [29].

We also evaluated the diagnostic performance of endoscopists and PolyDeep for the optical diagnosis of colorectal lesions during colonoscopy. In the clinical validation of the POLAR system, the optical diagnosis of diminutive colorectal lesions was assessed [30]. Although this was not the primary objective of our study, both endoscopists and PolyDeep performed optical diagnoses of diminutive lesions, with the mean polyp size in our study being 5.1 ± 4.9 mm [30]. In the POLAR study, lesions were classified as neoplastic or non-neoplastic, which did not allow a direct comparison of results since our endoscopists used the NICE classification. Nonetheless, the sensitivity achieved by endoscopists and the POLAR system (92.4% and 89.4%) was higher than that obtained in our study by endoscopists and PolyDeep (76.6% and 82.6%), although the results across both studies remain comparable for endoscopists and CADx systems [30]. In another study employing a similar setting, where endoscopists could not see the AI system’s output and a second observer managed the system for optical diagnosis of neoplastic and non-neoplastic lesions, sensitivities of 95.5% for less experienced endoscopists and 90.8% for experienced endoscopists were reported [31]. Although we did not evaluate less experienced endoscopists, the sensitivity of the experienced endoscopists in our study (76.6%) was lower than 90.8% reported for experienced endoscopists and significantly inferior to the 95.5% observed for non-experts [31]. Meanwhile, the AI system in that study achieved a sensitivity of 89.7% for characterising neoplastic and non-neoplastic lesions, while PolyDeep demonstrated a sensitivity of 91.5% in our study being higher and comparable [31]. These findings suggest that the performance of less experienced endoscopists improves with the assistance of AI systems in optical diagnoses, likely driven by a sense of competition with the AI system. This evidence highlights the potential value of such systems as training tools for novice endoscopists. However, this competitive advantage did not appear to apply when the same technology is used by experienced endoscopists.

During the development of the study, we found that the optical diagnosis made by PolyDeep was sometimes suboptimal. In this regard, the percentage of neoplasia displayed by PolyDeep on-screen fluctuates in a wide range, making it difficult to assess which is the real optical diagnosis of the CADx system. For this reason, we explored different alternatives to first improve the optical diagnosis and second ease the optical diagnosis task for endoscopists in the next randomised controlled trials (i.e., PolyDeep advance 2 and PolyDeep advance 3). This improvement involves the inclusion of specific symbols (i.e., loupe, waves as two brackets and hourglass) within the bounding box that show the percentage of neoplasia for optical diagnosis. When the percentage is displayed and the loupe icon appears, it means that the endoscopist needs to be closer to the polyp to obtain a more stable diagnosis. The wave symbol is displayed when the polyp is in focus but there is excessive movement, preventing the CADx system from providing a stable diagnosis. Finally, the hourglass symbol informs the endoscopists to wait to allow the CADx system to process enough frames to provide a stable diagnosis.

PolyDeep offers significant value in both specialised hospitals and those lacking advanced technology. Its installation in hospitals without an existing technological infrastructure can enhance the quality of colonoscopies. Additionally, implementing this technology benefits both specialised and non-specialised hospitals, particularly by supporting novice endoscopists.

Our study has several strengths: first, it was conducted in a real clinical setting, providing a valuable assessment of PolyDeep’s performance in real colonoscopy procedures. Second, it is innovative because the expert endoscopists were blinded to the CADe/x system’s output. Third, the diagnostic performance obtained for polyp detection is consistent with literature reports. Fourth, PolyDeep was built with a YOLOv3 detection network coupled with an object tracking algorithm, which allows linking the independent predictions made by YOLOv3 on each frame to specific polyps. By doing this, we can present the neoplasia probability of each polyp as the average of the probabilities calculated for that polyp during a pre-specified number of frames (50 frames in the final setting).

On the other hand, our study has several limitations: first, the study was not fully blinded to the endoscopists, as the PolyDeep image was visible on a separate screen at the back of the endoscopists in the room. The screen was not in the endoscopists’ direct line of sight, but this could have introduced cognitive bias due to the presence of a second observer and the CADe/x system monitoring the procedure. Potentially, this could lead to an artificial improvement in the endoscopists’ diagnostic performance. Second, our study did not measure the time taken for polyp detection between endoscopists and PolyDeep. As a result, we could not determine whether there was a delay in detection between both. Third, while low-quality colonoscopies can negatively impact the diagnostic performance of CAD systems, the quality of screening colonoscopies is generally well-controlled and high. In 197 valid colonoscopies, we obtained a mean overall Boston Bowel Preparation Scale score of 7.7 ± 1.34 and a mean withdrawal time, including instrumentation, of 14:27 ± 09:02. Fourth, PolyDeep was developed using the latest available detection technology at the time, specifically the YOLOv3 detection model. Since we began clinical validation, newer versions of YOLO have been released, including YOLOv8 in January 2023 and YOLOv11 in September 2024 [32]. As a result, it was not feasible to implement YOLOv11 in our system, but this remains a potential area for future improvement. To our knowledge, other currently available tools have also not been built using the latest YOLO version. Fifth, the study did not evaluate the impact of PolyDeep on colonoscopy quality indicators or relevant clinical outcomes, such as post-colonoscopy CRC, which is beyond our scope. Sixth, despite updates made prior to clinical validation, PolyDeep exhibited a high false-positive rate, which led to minor disruptions in the colonoscopy procedure. However, previous studies with other CADe systems, such as GI Genius CADe v2, reported only one to two false alarms per patient, which did not affect withdrawal time [33]. In our study, we reported 1.95 false alarms per patient. To overcome this problem, we could apply the solution at two main levels, one at the clinical level and the other at the algorithmic level [34]. At the colonoscopy level, an adequate insufflation of air eliminates wrinkled walls, and the use of simethicone can reduce the bubble disturbances [34]. Furthermore, antispasmodic agents contribute to a more relaxed colon, providing the algorithm with a clear video image [34]. At the PolyDeep algorithmic level, we could apply a median filter or a threshold time for persistent false alarm detection [14]. As another option, we could change the present algorithm (i.e., YOLOv3) into an active learning model, which reported a reduction in the false positive rate with regard to YOLOv3 [35]. Finally, the endoscopists classified lesions based on the NICE classification (i.e., they classified adenomas) and PolyDeep classified them based on the probability of detecting neoplasia. So, we could not compare the diagnostic performance for optical diagnosis of PolyDeep and the endoscopists.

In summary, our study demonstrated similar results to those found in other studies. Both endoscopists and PolyDeep showed comparable diagnostic performance for the detection of colorectal lesions. For optical diagnosis, both agents showed moderate performance. These findings highlight the potential of PolyDeep as an effective support tool in colonoscopy procedures.

## Figures and Tables

**Table 1:** Characteristics of the lesions detected.

		Both ^1^ *n* = 345 (%)	Only Endoscopists ^2^ *n* = 39 (%)	Only PolyDeep ^3^ *n* = 26 (%)
**Lesion type** ***n* = 410**	**Polyps (*n* = 343)**	294 (85.72%)	29 (8.45%)	20 (5.83%)
	**Adenoma (*n* = 231)**	201 (87.01%)	17 (7.36%)	13 (5.63%)
	**Serrated lesions (*n* = 39)**	37 (94.87%)	2 (5.13%)	0 (0%)
	**Hyperplastic lesions (*n* = 73)**	56 (76.71%)	10 (13.70%)	7 (9.59%)
	**Normal mucosa (*n* = 42)**	32 (76.19%)	6 (14.29%)	4 (9.52%)
	**Colorectal Cancer (*n* = 5)**	5 (100%)	0 (0%)	0 (0%)
	**Not histology (*n* = 20)**	14 (70.00%)	4 (20.00%)	2 (10.00%)
**Advanced lesions ^4^** ***n* = 385**	**Yes (*n* = 73)**	71 (97.26%)	1 (1.37%)	1 (1.37%)
	**No (*n* = 312)**	255 (81.73%)	34 (10.89%)	23 (7.37%)
**Size** ***n* = 385**	**≤5 mm (*n* = 289)**	234 (80.97%)	32 (11.07%)	23 (7.96%)
	**>5 mm (*n* = 96)**	92 (95.83%)	3 (3.13%)	1 (1.04%)
**Location** ***n* = 385**	**Right colon (*n* = 207) ^5^**	176 (85.02%)	20 (9.66%)	11 (5.32%)
	**Left colon (*n* = 178) ^6^**	150 (84.27%)	15 (8.43%)	13 (7.30%)
**Morphology** ***n* = 385**	**Pedunculated (*n* = 59)**	57 (96.61%)	1 (1.70%)	1 (1.69%)
	**Sessile (*n* = 218)**	187 (85.78%)	15 (6.88%)	16 (7.34%)
	**Slightly elevated (*n* = 84)**	69 (82.14%)	12 (14.29%)	3 (3.57%)
	**Flat (*n* = 24)**	13 (54.17%)	7 (29.17%)	4 (16.66%)

^1^ Detections made by the endoscopists and PolyDeep at the same time. ^2^ Detections only made by the endoscopists. ^3^ Detections only made by PolyDeep. ^4^ Adenoma ≥ 10 mm, high-grade dysplasia, tubulovillous or villous histology or serrated lesions with dysplasia or ≥10 mm. ^5^ Polyps detected between the caecum and the splenic flexure. ^6^ Polyps detected between the descendent colon and the rectum.

**Table 2:** Diagnostic performance of endoscopists and PolyDeep for polyp (adenoma, serrated or hyperplastic lesion) detection.

	Sensitivity (%) (95% CI)			Specificity (%) (95% CI)			PPV (%) (95% CI)		NPV (%) (95% CI)		Odds Ratio (95% CI)	
	Endoscopists	PolyDeep	P ^1^	Endoscopists	PolyDeep	P ^1^	Endoscopists	PolyDeep	Endoscopists	PolyDeep	Endoscopists	PolyDeep
**Polyp** **(*n* = 385)**	94.2 (91.0–96.3)	91.5 (87.9–94.1)	0.2	9.5 (3.1–23.6)	14.3 (5.9–29.2)	0.7	89.5 (85.7–92.3)	89.7 (85.9–92.6)	16.7 (5.5–38.2)	17.1 (7.2–34.3)	1.7 (0.4–5.45)	1.8 (0.6–4.8)
**Polyp ≤5 mm** **(*n* = 289)**	92.3 (88.1–95.2)	89.5 (84.8–92.9)	0.4	9.8 (3.1–24.1)	14.6 (6.1–29.9)	0.7	86.1 (81.2–89.9)	86.4 (81.4–90.2)	17.4 (5.7–39.5)	18.8 (7.9–37.1)	1.3 (0.3–4.2)	1.5 (0.5–4.0)
**Polyp > 5 mm** **(*n* = 96)**	98.9 (93.4–99.9)	96.8 (90.4–99.2)	0.6	0.0 (10.8–94.5)	0.0 (10.8–94.5)	^6^	98.9 (93.4–99.9)	98.9 (93.3–99.9)	0.0 (10.8–94.5)	0.0 (3.18–69.0)	0.0 (0.0–3502.3)	0.0 (0.0–1186.6)
**Right colon ^2^** **(*n* = 207)**	95.0 (90.4–97.5)	91.1 (85.7–94.6)	0.2	7.1 (1.3–25.0)	14.3 (4.7–33.6)	0.7	86.7 (81.0–91.0)	87.2 (81.3–91.4)	18.2 (3.2–52.3)	20.0 (6.6–44.3)	1.4 (0.1–7.6)	1.7 (0.4–5.9)
**Left colon ^3^** **(*n* = 178)**	93.3 (88.0–96.4)	92.1 (86.5–95.5)	0.8	14.3 (2.5–43.9)	14.3 (2.5–43.8)	1.0	92.7 (87.4–96.0)	92.6 (87.2–96.0)	15.4 (2.7–46.3)	13.3 (2.3–41.6)	2.3 (0.2–12.6)	1.9 (0.2–10.2)
**Protruded lesions ^4^** **(*n* = 277)**	94.0 (90.2–96.5)	94.8 (91.1–97.1)	0.9	8.0 (1.4–27.5)	12.0 (3.2–32.3)	1.0	91.2 (86.9–94.2)	91.6 (87.4–94.5)	11.8 (2.1–37.8)	18.8 (5.0–46.3)	1.4 (0.1–6.5)	2.5 (0.4–10.1)
**Non-Protruded ^5^** **(*n* = 108)**	94.5 (87.1–98.0)	82.4 (72.7–89.3)	<0.05	11.8 (2.1–37.8)	17.6 (4.7–44.2)	1.0	85.2 (76.4–91.2)	84.3 (74.7–90.8)	28.6 (5.1–69.7)	15.8 (4.2–40.5)	2.3 (0.2–15.5)	1.0 (0.2–4.2)

^1^ Differences are compared using the McNemar test. A *p*-value < 0.05 is considered statistically significant. ^2^ The right colon corresponds to caecum, ascendent colon, hepatic flexure, transverse colon, splenic flexure. ^3^ The left colon includes descendent colon, sigmoid colon and rectum. ^4^ Protruded lesions include all the lesions with morphology pedunculated and sessile. ^5^ Non-protruded lesions include lesions slightly elevated, flat and depressed lesions. ^6^ Cells without values mean that we cannot calculate this value. Abbreviations: PPV, positive predictive value; NPV, negative predictive value; CI, confidence interval.

**Table 3:** Diagnostic performance for polyp detection for PolyDeep and endoscopists according to histology.

	Sensitivity (%) (95% CI)			Specificity (%) (95% CI)			PPV (%) (95% CI)		NPV (%) (95% CI)		Odds Ratio (95% CI)	
	Endoscopists	PolyDeep	P ^1^	Endoscopists	PolyDeep	P ^1^	Endoscopists	PolyDeep	Endoscopists	PolyDeep	Endoscopists	PolyDeep
**Neoplasia ^2^** **(*n* = 385)**	95.2 (91.7–97.3)	92.9 (89.0–95.6)	0.3	9.6 (5.1–16.8)	13.9 (8.4–21.9)	0.4	71.2 (66.1–75.7)	71.7 (66.6–76.3)	45.7 (26.2–66.8)	45.7 (29.2–63.1)	2.1 (0.8–5.2)	2.1 (1.0–4.6)
**Adenoma** **(*n* = 385)**	94.4 (94.3–96.8)	92.6 (88.3–95.5)	0.5	7.1 (3.8–12.7)	11.7 (7.3–18.1)	0.2	60.4 (55.1–65.4)	61.1 (55.8–66.2)	45.8 (26.2–66.8)	51.4 (34.3–68.3)	1.3 (0.5–3.2)	1.7 (0.8–3.6)
**Serrated lesions** **(*n* = 385)**	100.0 (88.8–99.8)	94.9 (81.4–99.1)	^-^	6.9 (4.5–10.3)	9.5 (6.8–13.3)	0.3	10.8 (7.9–14.6)	10.6 (7.6–14.4)	100.0 (82.8–99.6)	94.3 (79.5–99.0)	^-^	2.0 (0.5–17.4)
**Advanced lesions ^3^** **(*n* = 73)**	98.6 (91.6–99.9)	98.6 (91.6–99.9)	1.0	^-^	^-^	^-^	^-^	^-^	^-^	^-^	^-^	^-^

^1^ Differences are compared using the McNemar test. A *p*-value < 0.05 is considered statistically significant. ^2^ Adenoma, serrated lesions. ^3^ Adenomas or serrated lesions ≥10 mm, adenomas with villous histology or high-grade dysplasia or serrated lesions with dysplasia. Abbreviations: PPV, positive predictive value; NPV, negative predictive value; CI, confidence interval.

## Data Availability

The raw data supporting the conclusions of this article will be made available by the authors on request.

## References

[1] Lopes S.R., Martins C., Santos I.C., Teixeira M., Gamito É., Alves A.L. (2024). Colorectal Cancer Screening: A Review of Current Knowledge and Progress in Research. World J. Gastrointest. Oncol..

[2] Kudo S.E., Mori Y., Abdel-Aal U.M., Misawa M., Itoh H., Oda M., Mori K. (2021). Artificial Intelligence and Computer-Aided Diagnosis for Colonoscopy: Where Do We Stand Now?. Transl. Gastroenterol. Hepatol..

[3] Zhao L., Wang N., Zhu X., Wu Z., Shen A., Zhang L., Wang R., Wang D., Zhang S. (2024). Establishment and Validation of an Artificial Intelligence-Based Model for Real-Time Detection and Classification of Colorectal Adenoma. Sci. Rep..

[4] Young E., Edwards L., Singh R. (2023). The Role of Artificial Intelligence in Colorectal Cancer Screening: Lesion Detection and Lesion Characterization. Cancers.

[5] Maas M.H.J., Rath T., Spada C., Soons E., Forbes N., Kashin S., Cesaro P., Eickhoff A., Vanbiervliet G., Salvi D. (2024). A Computer-Aided Detection System in the Everyday Setting of Diagnostic, Screening, and Surveillance Colonoscopy: An International, Randomized Trial. Endoscopy.

[6] Lecun Y., Bengio Y., Hinton G. (2015). Deep Learning. Nature.

[7] U.S. Food and Drug Administration Artificial Intelligence and Machine Learning (AI/ML)-Enabled Medical Devices. https://www.fda.gov/medical-devices/software-medical-device-samd/artificial-intelligence-and-machine-learning-aiml-enabled-medical-devices.

[8] Liang F., Wang S., Zhang K., Liu T.-J., Li J.-N. (2022). Development of Artificial Intelligence Technology in Diagnosis, Treatment, and Prognosis of Colorectal Cancer. World J. Gastrointest. Oncol..

[9] Kamitani Y., Nonaka K., Isomoto H. (2022). Current Status and Future Perspectives of Artificial Intelligence in Colonoscopy. J. Clin. Med..

[10] Zhao S., Wang S., Pan P., Xia T., Chang X., Yang X., Guo L., Meng Q., Yang F., Qian W. (2019). Magnitude, Risk Factors, and Factors Associated with Adenoma Miss Rate of Tandem Colonoscopy: A Systematic Review and Meta-Analysis. Gastroenterology.

[11] Wang P., Liu X.-G., Kang M., Peng X., Shu M.-L., Zhou G.-Y., Liu P.-X., Xiong F., Deng M.-M., Xia H.-F. (2023). Artificial Intelligence Empowers the Second-Observer Strategy for Colonoscopy: A Randomized Clinical Trial. Gastroenterol. Rep..

[12] Sinonquel P., Sinonquel P., Eelbode T., Eelbode T., Pech O., Pech O., De Wulf D., De Wulf D., Dewint P., Dewint P. (2024). Clinical Consequences of Computer Aided Colorectal Polyp Detection. Gut.

[13] Sinonquel P., Eelbode T., Hassan C., Antonelli G., Filosofi F., Neumann H., Demedts I., Roelandt P., Maes F., Bisschops R. (2021). Real-time Unblinding for Validation of a New CADe Tool for Colorectal Polyp Detection. Gut.

[14] Nogueira-Rodríguez A., Glez-Peña D., Reboiro-Jato M., López-Fernández H. (2023). Negative Samples for Improving Object Detection—A Case Study in AI-Assisted Colonoscopy for Polyp Detection. Diagnostics.

[15] Nogueira-Rodríguez A., Reboiro-Jato M., Glez-Peña D., López-Fernández H. (2022). Performance of Convolutional Neural Networks for Polyp Localization on Public Colonoscopy Image Datasets. Diagnostics.

[16] Davila-Piñón P., Nogueira-Rodríguez A., Díez-Martín A.I., Codesido L., Herrero J., Puga M., Rivas L., Sánchez E., Fdez-Riverola F., Glez-Peña D. (2024). Optical Diagnosis in Still Images of Colorectal Polyps: Comparison between Expert Endoscopists and PolyDeep, a Computer-Aided Diagnosis System. Front. Oncol..

[17] Nogueira-Rodríguez A., Domínguez-Carbajales R., Campos-Tato F., Herrero J., Puga M., Remedios D., Rivas L., Sánchez E., Iglesias Á., Cubiella J. (2022). Real-Time Polyp Detection Model Using Convolutional Neural Networks. Neural Comput. Appl..

[18] Nogueira Rodríguez A. (2022). Deep Learning Techniques for Computer-Aided Diagnosis in Colorectal Cancer. Ph.D. Thesis.

[19] PolyDeep Research Consortium Colorectal Polyp Image Cohort (PIBAdb). https://www.iisgaliciasur.es/home/biobanco/cohorte-pibadb/.

[20] Rex D.K. (2009). Narrow-Band Imaging Without Optical Magnification for Histologic Analysis of Colorectal Polyps. Gastroenterology.

[21] Parsa N., Rex D.K., Byrne M.F. (2021). Colorectal Polyp Characterization with Standard Endoscopy: Will Artificial Intelligence Succeed Where Human Eyes Failed?. Best Pract. Res. Clin. Gastroenterol..

[22] Gangwani M.K., Haghbin H., Ishtiaq R., Hasan F., Dillard J., Jaber F., Dahiya D.S., Ali H., Salim S., Lee-Smith W. (2024). Single Versus Second Observer vs. Artificial Intelligence to Increase the ADENOMA Detection Rate of Colonoscopy—A Network Analysis. Dig. Dis. Sci..

[23] Sinonquel P., Eelbode T., Pech O., De Wulf D., Dewint P., Neumann H., Antonelli G., Tate D., Lemmers A., Pilonis N. (2023). Clinical validation of a computer-aided detection model for colorectal polyp detection (cad-artipod) trial using a second observer and real-time unblinding. Gastrointest. Endosc..

[24] Lee C.K., Park D.I., Lee S.-H., Hwangbo Y., Eun C.S., Han D.S., Cha J.M., Lee B.-I., Shin J.E. (2011). Participation by Experienced Endoscopy Nurses Increases the Detection Rate of Colon Polyps during a Screening Colonoscopy: A Multicenter, Prospective, Randomized Study. Gastrointest. Endosc..

[25] Buchner A.M., Shahid M.W., Heckman M.G., Diehl N.N., McNeil R.B., Cleveland P., Gill K.R., Schore A., Ghabril M., Raimondo M. (2011). Trainee Participation Is Associated with Increased Small Adenoma Detection. Gastrointest. Endosc..

[26] Mangas-Sanjuan C., De-Castro L., Cubiella J., Díez-Redondo P., Suárez A., Pellisé M., Fernández N., Zarraquiños S., Núñez-Rodríguez H., Álvarez-García V. (2023). Role of Artificial Intelligence in Colonoscopy Detection of Advanced Neoplasias: A Randomized Trial. Ann. Intern. Med..

[27] Zhang H., Wu Q., Sun J., Wang J., Zhou L., Cai W., Zou D. (2023). A Computer-Aided System Improves the Performance of Endoscopists in Detecting Colorectal Polyps: A Multi-Center, Randomized Controlled Trial. Front. Med..

[28] Maas M.H.J., Soons E., Lebwohl B., Lewis S.K., Ngamruengphong S., Landsman M.J., Neumann H., Shirin H., Katz L.H., Benson A.A. (2024). A Computer-Aided Polyp Detection System in Screening and Surveillance Colonoscopy: An. International, Multicentre, Randomised, Tandem Trial. Lancet Digit. Health.

[29] Mori Y., Jin E.H., Lee D. (2023). Enhancing Artificial Intelligence Doctor Collaboration for Computer-Aided Diagnosis in Colonoscopy through Improved Digital Literacy. Dig. Liver Dis..

[30] Houwen B.B.S.L., Hazewinkel Y., Giotis I., Vleugels J.L.A., Mostafavi N.S., van Putten P., Fockens P., Dekker E. (2022). Computer-Aided Diagnosis for Optical Diagnosis of Diminutive Colorectal Polyps Including Sessile Serrated Lesions: A Real-Time Comparison with Screening Endoscopists. Endoscopy.

[31] Baumer S., Streicher K., Alqahtani S.A., Brookman-Amissah D., Brunner M., Federle C., Muehlenberg K., Pfeifer L., Salzberger A., Schorr W. (2023). Accuracy of Polyp Characterization by Artificial Intelligence and Endoscopists: A Prospective, Non-Randomized Study in a Tertiary Endoscopy Center. Endosc. Int. Open.

[32] Jegham N., Koh C.Y., Abdelatti M., Hendawi A. (2024). Evaluating the Evolution of YOLO (You Only Look Once) Models: A Comprehensive Benchmark Study of YOLO11 and Its Predecessors. arXiv.

[33] Cherubini A., Dinh N.N. (2023). A Review of the Technology, Training, and Assessment Methods for the First Real-Time AI-Enhanced Medical Device for Endoscopy. Bioengineering.

[34] Hsieh Y.-H., Tang C.-P., Tseng C.-W., Lin T.-L., Leung F.W. (2021). Computer-Aided Detection False Positives in Colonoscopy. Diagnostics.

[35] Guo Z., Zhang R., Li Q., Liu X., Nemoto D., Togashi K., Niroshana S.I., Shi Y., Zhu X. Reduce False-Positive Rate by Active Learning for Automatic Polyp Detection in Colonoscopy Videos. Proceedings of the 2020 IEEE 17th International Symposium on Biomedical Imaging (ISBI).

